# The financial impact and utilization of inpatient dermatology services: historical insights and future implications

**DOI:** 10.1007/s00403-025-03867-y

**Published:** 2025-02-08

**Authors:** Olivia Burke, Mara Hartoyo, Rachel Lin, Robert S. Kirsner, Scott A. Elman

**Affiliations:** https://ror.org/02dgjyy92 grid.26790.3a 0000 0004 1936 8606 Dr. Phillip Frost Department of Dermatology and Cutaneous Surgery, University of Miami Miller School of Medicine, 1600 NW 10th Ave RMSB Building, Miami, FL 33136 USA

**Keywords:** Inpatient dermatology, Complex medical dermatology, Reimbursement, Cost-effectiveness

## Abstract

Skin diseases affect millions of Americans, imposing a large financial burden on the U.S. healthcare system annually. Inpatient dermatology is a subspecialty focused on treating complicated skin diseases in hospitalized patients. Utilization of these services enhances diagnostic accuracy, shorten hospital stays, lower readmission rates, and improve patient outcomes. However, studies have indicated an overall decline in inpatient dermatology consultations and dermatology as primary admitting services. Currently, only two academic hospitals in the United States grant dermatologists admitting privileges, indicating decreased exposure to inpatient dermatology in residency despite the need for more hospital-based dermatologists. Therefore, this narrative review aims to characterize the financial impact and utilization of inpatient dermatology services. Historical and recent data consistently highlight the financial benefit of dermatologic hospitalizations and poor utilization of inpatient dermatology consultations. Teledermatology consultations also improve diagnostic accuracy and expedite interventions to improve patient outcomes. However, challenges like reduced reimbursement, lack of protocols, and limited resident training in inpatient dermatology have discouraged dermatologists from providing inpatient consultations. Policy changes are needed to promote these services that benefit patients as well as health systems.

## Introduction

Skin diseases are among the most common health issues in the U.S., affecting millions of individuals each year [1]. This high prevalence translates to significant healthcare utilization. Over 600,000 hospital admissions each year are attributed to skin conditions, which include infections, inflammatory diseases, drug reactions, and neoplasms [2]. Dermatologic consultation is pivotal in these cases, influencing diagnosis and treatment in more than 60% of patients and improving outcomes [3–7].

The financial burden of inpatient skin conditions is substantial, exceeding $5 billion annually, accounting for approximately 0.5% of total inpatient healthcare expenditures [2, 8]. Hospitalizations primarily for skin diseases cost an average of $7,949 per admission, but this cost nearly doubles to $15,967 when skin conditions are comorbid with other diagnoses. Similarly, the length of stay is longer for comorbid conditions (7.3 days vs. 4.6 days) [9] Readmissions due to skin diseases further strain resources, adding over $1 billion annually to healthcare costs [9].

Historically, dermatologists often served as primary admitting physicians for patients with severe skin conditions. This inpatient dermatology model allowed for comprehensive and continuous dermatologic care for hospitalized patients, ensuring that complex cases received the specialized attention required. However, over the past few decades, changes in reimbursement models and the shift toward hospitalist care have significantly altered this dynamic [10]. The Society of Dermatology Hospitalists (SDH) proactively advocates for a greater role for dermatologists in inpatient care through a new inpatient *consultative* role [11]. This inpatient consultative model can sometimes lead to fragmented care, where the nuances of dermatologic conditions may not be fully integrated into the overall management plan. In contrast, a few institutions, where dermatologists act as both consultants and primary admitting physicians, highlight the benefits of integrated dermatologic care in hospitals. This review examines the clinical and financial implications of inpatient dermatology by synthesizing current literature on its utilization, trends, and impact.

## Methods

This narrative review synthesizes information from a range of sources, including peer-reviewed journal articles, retrospective cohort studies, and cross-sectional studies. Relevant literature was identified through searches in PubMed using keywords such as “inpatient dermatology”, “dermatology consult, “hospital dermatology”, “finance”, and “economics”. The review focuses on studies published in the past two decades to ensure a contemporary understanding of the topic. Articles were selected based on their relevance to the financial and clinical impact of inpatient dermatology services, as well as their contribution to understanding trends and challenges in this field.

## Discussion

### Financial implications of the suboptimal consultative model for inpatient dermatology

Hospitalizations for dermatologic diseases are a major burden to the healthcare system, which necessitates improvement in the continuity of treatment between hospitalists and consulting dermatologists. A 2014 study found that hospitalizations primarily for skin disease accounted for 2.01% of all admissions, costing $5.04 billion. Additionally, 12.1% of hospitalized patients were diagnosed with primary or comorbid skin disease, with bacterial infections being the most common (mean admission cost $7,024), followed by ulcers ($12,984) and connective tissue disorders ($15,577) [2].

The financial burden extends to pediatric patients, with dermatologic conditions comprising 4.2% of pediatric admissions and costing $379.8 million in 2012. The highest costs were associated with cutaneous lymphomas ($58,294 per admission), congenital skin abnormalities ($24,186), and ulcers ($17,064). These disparities underscore the need to improve access to outpatient dermatologic care and restructure the consultative model to provide comprehensive inpatient dermatologic care for underserved populations [12]. There are significant racial and socioeconomic disparities in pediatric dermatology hospitalization, which suggests the need to expand access to outpatient dermatologic care. Additionally, it may be necessary to restructure the suboptimal inpatient dermatology consultative model to provide more congruent inpatient dermatologic care to patients who cannot access outpatient services.

Readmission rates highlight the inefficiencies of the current model. A study of 3.6 million hospitalizations revealed 9.8% resulted in all-cause readmissions within 30 days, with 3.3% readmitted for the same diagnosis, incurring a mean cost of $8,995 per readmission. This study underscores the substantial burden of readmissions for dermatologic diseases and emphasizes the importance of implementing dermatologists as a primary admitting team, as opposed to just a consulting team, to reduce associated morbidity and costs [13].

Patients hospitalized with severe skin disease may benefit from an expanded and more integrated healthcare team including not only primary admitting inpatient dermatologists but also palliative care specialists. The mortality rate for patients hospitalized principally for skin disease was estimated to be 0.47% [2]. However, A retrospective investigation aimed at characterizing the use of palliative care for patients hospitalized with severe skin disease reported that in a sample of 193 inpatient dermatology consultations, palliative care was consulted in only 5.7% of cases. This study suggests the consultative model for palliative care for patients with severe dermatologic disease also results in suboptimal and fragmented care [14]. The substantial costs to the healthcare system and high readmission rates as reported by this review suggests the need for implementation of primary admitting inpatient dermatologists, instead of consulting dermatologists, to improve treatment and outcomes of patients hospitalized for skin disease.

### Optimizing inpatient dermatology

The current model of inpatient dermatology consultations remains underutilized. In 2017, dermatologists were consulted in only 4.6% of skin-related hospitalizations in the Medicare population, which equates to approximately 8,867 consultations per year [15]. This small consultation rate achieved $19.0 million to $38.3 million in Medicare cost savings in 2017 alone. It was estimated that if the dermatology consultation rate was increased to 10% of skin-related hospitalizations, Medicare cost savings could increase to $41.3 to $83.3 million [15]. To increase this rate to 10%, we would need to conduct an additional 10,402 consultations annually, bringing the total to about 19,269. To reach 20%, an additional 29,671 consultations would be necessary, totaling 38,538 per year. In terms of staffing, we estimate that one dermatologist can handle about 500 inpatient consultations annually (2–3 consults per workday excluding weekends, holidays, and vacation days). To meet the 10% target, this would require 21 additional dermatologists. To reach 20%, about 60 more dermatologists would be needed. Expanding inpatient consultations could reduce dermatologists’ availability for outpatient visits, potentially creating a trade-off between the two services. To address this potential trade-off, several strategies could be considered. By involving residents and fellows more deeply in inpatient care, we can distribute the workload more evenly and ensure that both inpatient and outpatient services are adequately staffed. In addition, exposure to inpatient dermatology as at the resident level may inspire a greater interest in this subspecialty, potentially addressing the shortage of physicians willing to perform inpatient consultations [16].

Improving the financial incentives for inpatient consultations could play a crucial role in encouraging more dermatologists to engage in this aspect of care [17]. Advocacy efforts through professional organizations and lobbying for Medicare/Medicaid reimbursement changes can set the stage for higher payments.

Alternatively, inpatient dermatology, where dermatologists are integrated as part of the inpatient care team rather than only being consulted, could provide more timely and comprehensive care. This model is associated with increased diagnostic and therapeutic accuracy, and significantly lower risk-adjusted 30-day mortality and 30-day readmissions [18]. In addition, inpatient dermatology addresses the median 2-day lag time between hospitalization and time of consultation associated with consultative model [7]. Notably, this model may achieve improved outcomes through higher rates of outpatient dermatology follow-up, partially offsetting the tradeoff discussed earlier [19]. Expanding upon such data-driven research demonstrating the value of inpatient dermatology, can further bolster advocacy efforts for reimbursement.

### Benefit of teledermatology filling the gaps

Implementation of teledermatology within the hospital setting may increase dermatologists’ reach and increase consultation availability without requiring bedside presence. A retrospective cohort study analyzing 1,320 inpatient encounters found that teledermatology consultations led to a change in diagnosis in 89.3% of suspected cellulitis cases, resulting in 87.7% of patients discontinuing unnecessary intravenous antibiotics. Additionally, teledermatology identified a need for skin biopsies in 36% of cases, which were subsequently performed [20]. Another study found that therapeutic agreement was higher between inpatient dermatologists and teledermatologists (77.4%) than hospitalists and dermatologists (58.5%) [21]. These findings highlight the diagnostic accuracy and improved patient outcomes associated with inpatient teledermatology.

Beyond inpatient settings, teledermatology has proven effective in the emergency department (ED). A retrospective analysis of 450 ED dermatology consultations found that teledermatology consultations altered diagnoses and treatment plans in 83% of cases. Follow-up evaluations with dermatology demonstrated high concordance with teledermatology (88%) [22]. Teledermatology’s utility becomes particularly apparent in underserved areas or institutions with limited access to dermatologists. Many dermatology clinics are located off-site from hospitals, requiring significant travel time for consultations. Teledermatology eliminates these delays, allowing timely interventions. In cases where a biopsy is deemed necessary by the consulting dermatologist, a physician assistant or nurse practitioner could perform the procedure under appropriate protocols, optimizing efficiency while addressing resource constraints. This approach may be particularly valuable in resource-limited settings.

Despite these advantages, barriers to widespread teledermatology implementation persist. These include non-standardized reimbursement schemes, lack of reimbursement for televisits in certain contexts—exacerbated by Medicare’s recent reduction in telehealth coverage—and the increased cost of teledermatology compared to usual care [23]. Protected time for teledermatology consultations is also limited [24]. Despite the cost of teledermatology being slightly higher than usual care, teledermatology drastically reduces wait time to initial definitive diagnosis, from 137.5 days for usual care and 50 days for teledermatology patients [25]. Therefore, teledermatology can be an avenue of providing earlier intervention for patients, which in certain conditions may improve prognosis.

Overall, most dermatologists support the integration of teledermatology into their inpatient duties. A survey reported that during the COVID-19 pandemic, the number of dermatologists using telehealth for inpatient consults rose from 40 to 90% [23]. Prior to the pandemic, only one surveyed provider reported a formal institutional protocol for conducting inpatient teledermatology, which rose to 77.8% of institutions following the pandemic. More importantly, provider attitudes regarding teledermatology has favorably shifted—substantial interest in adopting this technology was expressed by 85.7% of respondents from institutions without inpatient teledermatology, underscoring its perceived benefits in improving inpatient care accessibility [24]. Wider adoption of teledermatology can be another avenue of increasing inpatient dermatologists’ presence, particularly in regions with shortages of dermatologists. Addressing barriers such as reimbursement limitations and standardizing protocols will be critical to maximizing its potential, especially as health systems navigate Medicare’s reduced coverage of televisits.

### Reimbursement structures

Currently, reimbursement models in dermatology heavily favor high-margin fields like Mohs surgery, cosmetics, and dermatopathology, where procedures are both highly specialized and financially rewarding. Medicare payment data indicates that Mohs micrographic surgery (MMS) increased by 700% from 1992 to 2009, with Medicare payments for MMS averaging $424 for first-stage Mohs on the head, neck, hands, feet, or genitalia [26]. For MMS, often performed in a physician’s office, the non-facility fee based on the HCPCS code 17,311 ranges from $607 to $889, with work relative value units (RVU) at 6.20. In contrast, inpatient dermatologic care, especially consultative services, currently offers lower financial incentives. For example, for an incision and drainage of the skin and subcutaneous tissue, the most common procedure performed by inpatient dermatologists, the facility price based on the HCPCS 10,061 ranges from $167 to $229 with work RVU at only 2.45 [27].

Dermatologists have also pointed out that reimbursement rates have not kept pace with inflation. For example, in-facility reimbursement for HCPCS 10,061 has increased from $149.56 in 2007 to $182.06 in 2024—yet after adjusting for inflation, the appropriate reimbursement should be $227.03 [28]. This highlights not only the existing disparity in reimbursement for inpatient dermatology services compared to other dermatologic specialties, but also how this gap is further compounded by inflation.

## Discussion

Inpatient dermatology consultations have the potential to improve patient outcomes and reduce financial burdens, but as discussed in this review, the consultative model has shown to be underutilized, fragmented, and inadequate. Over 12% of hospitalized patients are diagnosed with skin disease, and related hospitalizations cost healthcare systems billions of dollars [2]. Additionally, around 10% of the hospitalizations are associated with dermatological conditions resulting in readmissions within 30 days. Dermatology consultation has the potential to reduce financial burden and improve patient outcomes by changing diagnosis and treatment in the majority of consulted patients [3–7, 22], but dermatology consultation is still significantly underutilized, with dermatologists only being consulted on 4.6% of skin-related hospitalizations [15]. Direct and indirect costs of fragmented management of skin disease, including extended hospital stays, misdiagnoses, and readmissions result in substantial healthcare burden. Implementation of primary admitting inpatient dermatologists is essential to improving congruency of management of inpatient skin disease.

The inpatient dermatology consultative model remains underutilized [15], and we have seen a reduction in inpatient dermatology consults over the years, which may be a consequence of overall cost-saving efforts by hospitals and healthcare systems. Additionally, decreased reimbursement has potentially discouraged dermatologists from considering inpatient hospital consultations as part of their practice. The lack of standardized protocols or resources that integrate dermatologists as a specialty consult may contribute to an overall deficit of inpatient dermatology consultants. Therefore, a restructuring of the current consultative model is essential. A primary admitting inpatient dermatology service can provide financial incentive to dermatologists and standardization of inpatient dermatologic care that the consultative model cannot offer.

Studies have showcased an unmet demand for dermatologists, particularly in rural areas [29]. Demand for dermatological services is projected to exceed supply by 28% by 2036, despite a 12.45% increase in the dermatology workforce during the same timeframe [29]. The need for inpatient dermatological services is further complicated by an existing shortage of dermatologists. A detailed pilot program by Giesey et al. describes family medicine or internal medicine residents rotating on inpatient dermatology consultation services, with a community dermatologist as the attending. Close communication was conducted to see if the patient required inpatient evaluation from the community dermatologist, otherwise, the patient was seen outpatient after discharge. Since most hospitals are not teaching centers, the solution must extend beyond residents—partnerships between hospitalists and community dermatologists as per-diem or part-time consultants may help bridge this gap. Addressing barriers to inpatient dermatology, such as reimbursement rates, physician time, and institutional bureaucracy, is required to further incentivize this partnership [30].

Therefore, to it is important to promote the utility of inpatient dermatology by quantifying the financial benefit and feasibility by determining reimbursement versus time spent. Unfortunately, literature on dermatological reimbursement as an outcome is minimal, often institution-dependent, and thereby difficult to generalize [27, 31]. No studies exist on the average consult length of inpatient dermatology consultation, but one study estimates 15.5–16.5 min are spent per outpatient dermatological visit [32]. One meta-analysis compared the economics of teledermatology to traditional consults, noting that teledermatology took on average 7.54 min longer and thereby had an average additional opportunity cost of €29.25 per consultation. However, two of the three studies mentioned were from 2000 to 2003, a different landscape from today’s high-speed internet, ubiquitous mobile devices, and advanced video technology [33, 34]. The most recent study from 2015 did not include reimbursement rates [35]. Newer studies to determine reimbursement versus time would help provide concrete numbers on the economic benefits of inpatient dermatology with or without teledermatology within current fee structures.

To address this issue and incentivize dermatologists to provide inpatient consultations, work RVUs should be adjusted to better reflect the cognitive effort, diagnostic complexity, and care coordination involved. RVUs for inpatient dermatology procedures would need to increase by at least 3.75 units to match the compensation for procedures like Mohs Micrographic Surgery (MMS). Additionally, implementing value-based care models that tie reimbursement to improved outcomes—such as reduced hospital stays and lower readmission rates—could optimize both patient care and dermatologist compensation. Lastly, dermatologists and the larger medical community should advocate for CPT code revisions that appropriately distinguish and reimburse higher-level inpatient consultations, particularly those involving complex diagnoses or extensive care coordination. However, alignment with payment benchmarks of other specialties is an important consideration.

Infectious disease physicians have dedicated inpatient time, allowing for inpatient consultation without RVU loss. For dermatologists, replacing clinic time with inpatient consultation translates directly to lost RVUs. A complex inpatient infectious disease consult generates only 3–4 work RVUs, similar to the 2.45 work RVUs generated by an incision and drainage procedure by an inpatient dermatologist [36]. In order to financially incentivize dermatologists to consult in the inpatient setting, work RVUs for inpatient procedures such as incision and drainage would need to be substantially higher than those allotted for inpatient infectious disease consultation, which would result in large financial discrepancies between dermatology and other specialists consulting on cases with similar complexity. Financially incentivizing an increase in inpatient dermatology, while also aligning payment models with the benchmarks of other specialties is a delicate balance. A solution to this would be increased implementation of physician assistants (PAs) and nurse practitioners (NPs) as inpatient consultants. This would allow for expansion of inpatient dermatologic care, while minimizing RVU loss, and aligning inpatient dermatology payment models with other inpatient consultants.

The transition from a consultative model to incorporating a primary admitting inpatient dermatology team may take time. Teledermatology has the potential to close the gap in managing inpatient skin disease during this transition. Studies have proven the efficacy of teledermatology, with over 90% of consults in concordance with inpatient dermatology consults [21]. This is particularly relevant in under-resourced areas that may not have the ability to staff an in-house primary admitting inpatient dermatologist. In this way, teledermatology can serve to improve health equity by expanding access to specialty care [12]. The use of teledermatology dramatically reduces the initial time to diagnosis, which has cost-saving and patient outcome benefits [25]. In addition, teledermatology can be a tool to train resident dermatologists in hospital medicine for programs that do not have a high volume of dermatological hospital patients.

### A call to action

Systemic change in government and healthcare protocols are necessary to implement inpatient dermatologists as a primary admitting team and standardize telehealth protocols in low-resource areas. Increased financial compensation for inpatient dermatologists and teledermatologists, as well as a more concrete focus on inpatient training in dermatology residency programs, is necessary to incentivize dermatologists to dedicate time to treating hospitalized patients. In order to optimize inpatient dermatology resident training within the current consultative model, both direct patient care learning, such as bedside teaching rounds, and nonpatient care activities such as case presentations and lectures, should be integrated into inpatient dermatology curricula [37]. Strategies used to facilitate successful consultations and interprofessional learning can be applied to dermatology residency training to promote interest and education for residents in inpatient care. These strategies include clear, concise communication, preparation of core questions, and confirming management steps for all parties [38]. Additionally, integrating inpatient teledermatology into residency training may be a viable option for institutions with limited in-person consultative capacity and no possibility of a primary dermatology admitting service. A survey study of dermatology residents showed that over 90% of residents reported teledermatology enhanced their residency education [39], suggesting teledermatology could be implemented to improve inpatient education, thereby promoting familiarity and confidence for dermatologists in the hospital setting.

Underlying these issues is also the current deficit of dermatologists needed to fulfill the need for basic dermatological services, let alone inpatient consultations. Additionally, most American hospitals are not teaching hospitals nor have inpatient dermatology consults. Therefore, change should not be bottlenecked at the residency level. Increasing reimbursement for community dermatologists who choose to provide inpatient services should be discussed to incentivize these services. These institutional and systemic changes are crucial as congruent inpatient dermatologic care will not only reduce health spending and improve patient outcomes, it may also serve to improve overall health equity.

## Data Availability

No datasets were generated or analysed during the current study.
